# Supplementary figures and images for: Cnot4 heterozygosity attenuates high fat diet-induced obesity in mice and impairs PPARγ-mediated adipocyte differentiation
Source: PLoS One. 2025 May 27;20(5):e0316417. doi: 10.1371/journal.pone.0316417 (PMC12111730; doi:10.1371/journal.pone.0316417)

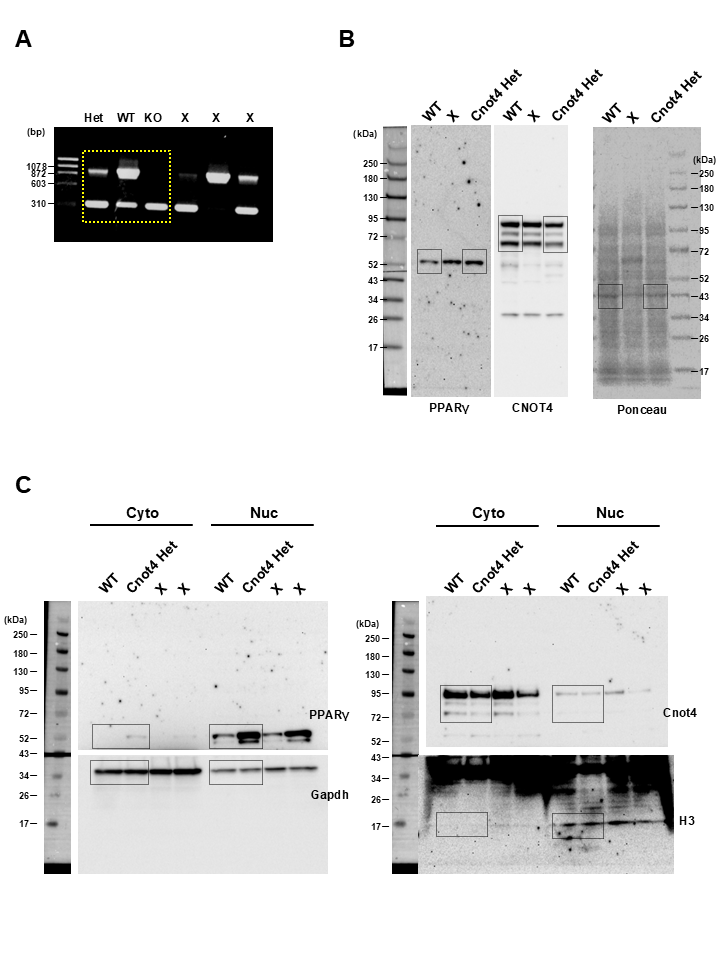

Supplement: S1 raw images — Yellow rectangle area is shown in the Figure. B, Uncropped images of the blot in Fig 5A. Black rectangle area is shown in the Figure. C, Uncropped images of the blot in Fig 5D. Black rectangle area is shown in the Figure. (TIF) [file pone.0316417.s001.TIF]
