# Supplementary material for: Cnot4 heterozygosity attenuates high fat diet-induced obesity in mice and impairs PPARγ-mediated adipocyte differentiation
Source: PLoS One. 2025 May 27;20(5):e0316417. doi: 10.1371/journal.pone.0316417 (PMC12111730; doi:10.1371/journal.pone.0316417)
Supplement: S1 Table — (DOCX) [file pone.0316417.s002.docx]

| genes | 5'-Sense-3' | 5'-Antisense-3' |
| --- | --- | --- |
| *Cnot4* | GAGGACTGTGCTCAGGTCAGCAGGCTCTTACA | GCCAGAATGCTCCCTCTGCTTCCTAACC |

**S1 Table. Primer sequences for genotyping**
