# Supplementary material for: Cnot4 heterozygosity attenuates high fat diet-induced obesity in mice and impairs PPARγ-mediated adipocyte differentiation
Source: PLoS One. 2025 May 27;20(5):e0316417. doi: 10.1371/journal.pone.0316417 (PMC12111730; doi:10.1371/journal.pone.0316417)
Supplement: S2 Table — (DOCX) [file pone.0316417.s003.docx]

| genes | 5'-Sense-3' | 5'-Antisense-3' |
| --- | --- | --- |
| *Cnot4* | GTGGAACCCACCGAAAGCTGAGAG | GCAAAGAGGGCATTCCACAGGG |
| *Srebf1a* | GATGTGCGAACTGGACACAGC | GAGAAGCTCTCAGGAGAGTTGG |
| *Srebf1c* | CGCGGACCACGGAGCCATG | GAGAAGCTCTCAGGAGAGTTGG |
| *Srebf2* | AGCCAAGGAGAGCCTGTACTG | GAGAGCGCACAGCTGCATCG |
| *Fasn* | GTTGGCCCAGAACTCCTGTA | GTCGTCTGCCTCCAGAGC |
| *Acaca* | CCGAAACTCCCAGAACTGCT | TCAGCTGCCTTCAGACCATC |
| *Hmgcs1* | TGGCACAGTACTCACCTC | CCTTCATCCAAACTGTGG |
| *Mvk* | ATATCCCTGGAGTGTGAGCG | CCACTGTGGCTTGCTCTAGA |
| *Adipoq* | CCGGAACCCCTGGCAG | CTGAACGCTGAGCGATACACA |
| *Pparg* | AACTCTGGGAGATTCTCCTGTTGA | TGGTAATTTCTTGTGAAGTGCTCATA |
| *aP2* | CACCGCAGACGACAGGAAG | GCACCTGCACCAGGGC |
| *Pdk4* | TTTTGCATTGTAGATGTTGTCCTT | TCAACCAATGTGGGAGTCAA |
| *Pgc1a* | GAAAGGGCCAAACAGAGAGA | GTAAATCACACGGCGCTCTT |
| *Ucp1* | GGCCTCTACGACTCAGTCCA | TAAGCCGGCTGAGATCTTGT |
| *Igfbp1* | GCCCAACAGAAAGCAGGAGATG | GTAGACACACCAGCAGAGTCCA |
| *Gdf15* | AGCTGCTACTCCGCGTCAA | GTAAGCGCAGTTCCAGCTG |
| *Fgf21* | ACCTGGAGATCAGGGAGGAT | CACCCAGGATTTGAATGACC |
| *Gapdh* | CTGCACCACCAACTGCTTAG | GTCTTCTGGGTGGCAGTGAT |
| ChIP *aP2* | GGGAGCCATAGAAGTCGCTC | CAGCCCTTCCTTGCCTTGTA |

**S2 Table. Primer sequences for qPCR**
